# Analysis of the Phenolic Compounds, Volatile Profile, and Evaluation of the Antioxidant Activity of 18 Different Varieties of Honey from the Italian Market

**DOI:** 10.3390/plants14193109

**Published:** 2025-10-09

**Authors:** Doaa Abouelenein, Laura Acquaticci, Eleonora Spinozzi, Agnese Santanatoglia, Gulzhan Khamitova, Ahmed M. Mustafa, Marco Cespi, Silvia Preziuso, Luca Bianchi, Filippo Maggi, Giovanni Caprioli

**Affiliations:** 1 Chemistry Interdisciplinary Project (ChIP) Research Center, School of Pharmacy, University of Camerino, Via Madonna Delle Carceri, 62032 Camerino, Italy; doaa.abouelenein@unicam.it (D.A.); laura.acquaticci@unicam.it (L.A.); eleonora.spinozzi@unicam.it (E.S.); agnese.santanatoglia@unicam.it (A.S.); gulzhan.khamitova@unicam.it (G.K.); ahmed.mustafa@unicam.it (A.M.M.); marco.cespi@unicam.it (M.C.); filippo.maggi@unicam.it (F.M.); 2 Research Group of Analytical Food Chemistry, National Food Institute, Technical University of Denmark (DTU), Kemitorvet 202, DK-2800 Lyngby, Denmark; 3 School of Biosciences and Veterinary Medicine, University of Camerino, Via Circonvallazione 93/95, 62024 Matelica, Italy; silvia.preziuso@unicam.it; 4 FAR Project Bee-Hon, School of Pharmacy, University of Camerino, 62032 Camerino, Italy; info@lucabianchi.net

**Keywords:** Italian honey, phenolic compounds, flavonoids, volatiles

## Abstract

The aim of this study was to present a comprehensive analysis of honey varieties from different botanical origins, focusing on their phenolic compounds’ composition, volatile profiles, and antioxidant activity. We simultaneously identified and quantified 37 bioactive compounds, including anthocyanins, flavonols, flavones, flavan-3-ols, proanthocyanidins, and phenolic acids, across various honey samples by HPLC-MS/MS. Total phenolic content (TPC), total flavonoid content (TFC), and antioxidant activity (AOA) were determined using UV-Vis spectrophotometric analysis. The content of phenolic compounds quantified by HPLC-MS/MS ranged from 19.56 to 243.94 mg·kg^−1^, highlighting a high presence of these antioxidant compounds (mainly phenolic acids), confirmed also by the positive correlation between TPC and DPPH values. Among volatiles compounds, analyzed by HS-SPME-GC-MS, benzene acetaldehyde and furfural resulted specific for two types of honey samples (H-7 and H-9), highlighting the possibility of searching for chemical markers to characterize honeys of different specie/origin. This study enhances our understanding of the bioactive potential of honey from different botanical origins and provides a foundation for future research on its health benefits.

## 1. Introduction

The research interest for honey is continuously increasing alongside its increasing global production [1]. In fact, the global production of honey was 1,850,868 tons in 2019, with China as the major producer, followed by Turkey and Argentina [2]. The European Union (EU), through the Council Directive 2001/110/EC, defined honey as “the natural sweet substance produced by *Apis mellifera* L. bees from the nectar of plants or from secretions of living parts of plants or excretions of plant-sucking insects on the living parts of plants, which the bees collect, transform by combining with specific substances of their own, deposit, dehydrate, store and leave in honeycombs to ripen and mature” [3]. Honey is composed mainly of sugars (80–85%), water (15–17%), and proteins (0.1–0.4%), with small amounts of enzymes, organic acids, vitamins, and phenolic compounds [4]. All these compounds are responsible for the physical and biochemical properties of honey, especially anti-inflammatory, antioxidant, antimicrobial, anticarcinogenic, and anti-diabetic, which give to this natural product a great potential as a health-promoter [5,6]. Physical, biochemical, and sensory properties are influenced by the botanical origin, the geographical area, flora, and entomological sources, as well as by processing factors. In fact, monofloral honey can be distinguished from multifloral honey on the base of botanical origin: monofloral honey is produced from the nectar of honeydew of a single botanical species or if its presence is the prevalent one, while the multifloral one comes from more than one botanical species [7]. Monofloral honey is characterized by distinguishing aromas, probably derived from the nectar, and it is considered a high-quality product; on the contrary, multifloral honey is composed by different nectars, coming from different flowers, and it should be composed by honey from sugar exudates (honeydew or forest honey) [8,9,10]. Phenolic compounds, including flavonoids and phenolic acids, are crucial constituents of honey, acting as natural indicators of its botanical origin and contributing significantly to its biological properties, particularly its antioxidant potential [11]. The rich phenolic content of honey, including flavonoids like apigenin, quercetin, and kaempferol, underpins its potential therapeutic uses, especially in cardiovascular disease treatment [12]. Numerous studies have shown that the texture, polyphenolic composition, and antioxidant, antibacterial, and radical-scavenging properties of honey vary based on its floral source and geographical origin. For instance, dark-coloured honeys generally contain higher levels of phenolic acids and flavonoids, leading to greater antioxidant capacity [13]. As a result, analyzing polyphenol content is crucial for assessing honey quality. Solid-phase extraction (SPE) is the most effective method for isolating these compounds from honey, with techniques employing polystyrene non-ionic sorbents like Amberlite XAD, ion-exchange resins such as Dowex 50WX8, or Bond Elut C18 cartridges [14,15]. Researchers have used SPE and high-performance liquid chromatography (HPLC) to quantify these compounds, revealing a wide range of concentrations depending on the honey’s floral source. In addition, several studies demonstrated that volatile compounds of honey can be used as fingerprints to determine the geographical origin and to expose any adulterations [16,17,18,19]. The most used technique to analyze volatile compounds in food was headspace-solid phase-microextraction (HS-SPME) coupled to gas chromatography–mass spectrometry (GC-MS), which uses a fibre to extract the volatiles in the headspace and the GC-MS to separate and identify all the compounds in the matrix. However, it is difficult to find specific chemical markers for honey [20,21] as the composition of honey is influenced not only by the botanical source, but also by geographical origin, harvesting season, storage conditions, possible interactions between chemical compounds in the honey that occur naturally, and also during thermal processing [22,23]. The colour of honey can vary from white to yellow and brown and it is influenced by the presence of phenolic compounds, flavonoids, and minerals [24], as well as by the monosaccharide content, which is responsible for sensorial and functional properties such as flavour, texture, moisture retention, shelf life, and conservation [4]. Given the high number of variables that influence the composition of honey, it is difficult to find criteria to determine the origin of the honey or detect any fraud; moreover, the literature was poor of studies on honeys from the Italian market. For these reasons, this study aims to conduct a comprehensive analysis of honey varieties from different botanical origins collected in the Italian market, focusing on their phenolic compound composition, volatile profiles, and antioxidant activity. Specifically, the research seeks to simultaneously identify and quantify 37 bioactive compounds, belonging to the chemical classes of anthocyanins, flavanols, flavones, flavan-3-ols, proanthocyanidins, and phenolic acids across different honey samples by HPLC-MS/MS. In addition, the study determined total phenolic content, flavonoid levels, and antioxidant activity using UV-Vis spectrophotometric analysis. Furthermore, the volatile profiles of the honey samples were determined using GC-MS, and the results were compared to provide detailed insights into their aromatic characteristics and the subtle nuances of their flavour profiles. The overall goal was to compare honey from multiple botanical origins in the Italian market to uncover how their chemical and antioxidant properties impact health benefits, enhancing our understanding of its bioactive potential and guiding future research.

## 2. Results

### 2.1. Spectrophotometric Analysis

#### 2.1.1. Total Phenolic Content (TPC)

The results obtained showed that the TPC (mg GAE/kg honey) varied among all honey samples, as shown in Table 1. TPC ranged from 176.18 to 868.41 mg GAE/kg. *Castanea sativa* honey resulted to have the highest TPC values (868.41 and 848.36 mg GAE/kg in H-1 and 8, respectively), followed by *Arbutus unedo* L. honey (761.11 mg GAE/kg in H-10). In addition, *Helianthus annuus* honey (H-11) and *Coriandrum sativum* L. honey (H-13) also had intermediate values (549.56 mg and 554.84 mg GAE/kg). *Robinia pseudoacacia* honey showed lower values (232.4 and 406.03 mg/kg) compared to other samples. While multifloral honey samples showed the lowest TPC (176.18 and 190.08 mg/kg in H-15 and 16, respectively).

#### 2.1.2. Total Flavonoid Content (TFC)

As shown in Table 1, TFC in honey samples varied from 36.15 to 307.90 mg RE/kg. The highest TFC value was found in *Helianthus annuus* honey (307.90 mg RE/kg in H-11), followed by *Arbutus unedo* L. honey (292.44 mg RE/kg in H-10) and *Coriandrum sativum* L. honey (242.69 mg RE/kg in H-13). As observed in TPC, TFC values of multifloral honey samples were different, ranging from 36.15 mg to 298.59 mg RE/kg in H-9 and 3, respectively. The highest value was found for multifloral honey (H-3) and it was higher than that of *Coriandru sativum* L. honey (H-13). The lowest TFC values were found in multifloral and *Robinia pseudoacacia* honey samples (H-2, 4, 9, 14, and 18).

#### 2.1.3. Antioxidant Activity (AOA)

**Figure 1:**
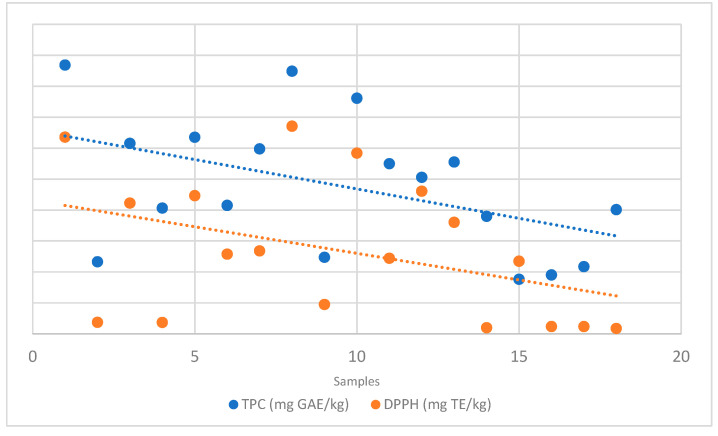
Relationship between TPC and DPPH values obtained from spectrophotometric analysis.

AOA was assessed through the DPPH assay. As shown in Figure 1, a positive correlation between TPC and AOA was observed, indicating that phenolic compounds are among the primary contributors to honey’s antioxidant capacity. The highest values were found in *Castanea sativa* honeys, which showed the highest TPC (635.54 and 670.92 mg TE/kg in H-1 and 8, respectively).

### 2.2. Colour Determination

**Figure 2:**
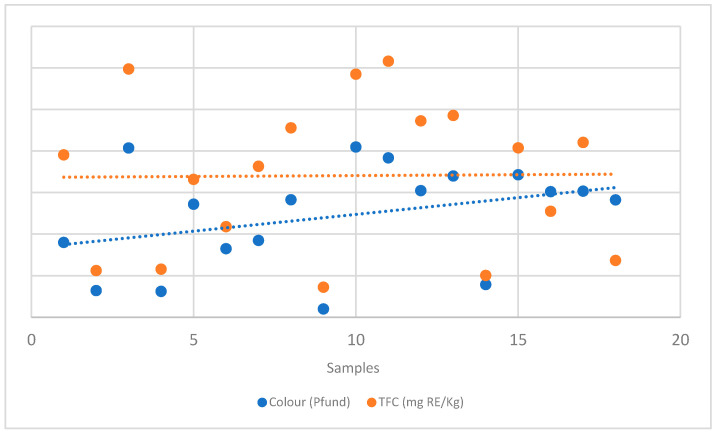
Relationship between TFC and PFUND values.

As shown in Table 2 and Figure S1, the colour of the various honey samples ranges from white to very dark amber. The lightest samples were *Robinia pseudoacacia* and blossom honey (H-2, 4, 9, and 14), the intermediates were *Castanea sativa* and multiflower honey (H-1, 6, and 7), while the others were all dark amber. Moreover, a positive correlation has also been found between TFC and colour (Pfund values in millimiters mm) in the tested honey samples, as shown in Figure 2.

### 2.3. Polyphenols Determination by HPLC-MS/MS

Among the 37 compounds quantified in the honey samples, 12 phenolic acids, 9 flavonols, 4 flavan-3-ols, 2 flavanones, and trans-cinnamic acid have been identified (Table S1). The content of all phenolic compounds ranges from 19.56 to 243.94 mg·kg^−1^ in multifloral honey (H-9) and *Coriandrum sativum* L. honey (H-13), respectively.

Phenolic acids were the major class in all honey samples, including both benzoic (gallic, 4&3-hydroxybenzoic, vanillic, syringic and ellagic acids) and cinnamic (neochlorogenic, chlorogenic, caffeic, *p*-coumaric, ferulic and dicaffeoylquinic acids) acid derivatives. Interestingly, *Coriandrum sativum* L. honey showed the highest phenolic acid content (230.05 mg·kg^−1^ in H-13), followed by multifloral honey samples (H-12, 16, 17, and 18). Vanillic acid was the most abundant phenolic compound found in most of the honey samples (ranging from 6.80 to 119.73 mg·kg^−1^ in all samples), while it was not detected in *Helianthus annuus* honey sample (H-11). 4-Hydroxy benzoic, 3-hydroxy benzoic, caffeic, and *p*-coumaric acids were also detected in significant amounts. *Helianthus annuus* and *Arbutus unedo* L. honey samples showed 4-hydroxy benzoic acid as the major phenolic acid. On the other hand, linden honey had *p*-coumaric acid as the major one. The lowest content of phenolic acids was found in blossom honey sample H-9 (18.64 mg·kg^−1^), with a high concentration of vanillic acid (7.96 mg·kg^−1^), while that of caffeic acid was very low (0.58 mg·kg^−1^) with respect to that of the other samples.

Regarding flavonoids group, anthocyanins, flavonols, flavan-3-ols, dihydrochalcones, and flavanones have been identified and quantified in honey samples. In particular, flavonols resulted in being the predominant class, followed by flavan-3-ols, flavanones, and anthocyanins, while dihydrochalcones were not present in honey samples. Among the flavonols, quercetin resulted in being the predominant compound in all honey samples, ranging from 0.08 to 9.62 mg·kg^−1^ and from 0.13 to 3.85 mg·kg^−1^, respectively. Except for *Castanea sativa*, *Robinia pseudoacacia*, and linden honey samples (H-1, 4, and 6) showed isorhamnetin as the major flavonoid. Among the flavan-3-ols, epicatechin and procyanidin A2 resulted in being the predominant in all honey samples, ranging from 0.01 to 0.87 mg·kg^−1^ and from 0.01 to 0.03 mg·kg^−1^, respectively. In flavanones, the most abundant compound was naringin, which is present in H-3, H-4, H-7, H-8, H-10, and H-18, with values from 0.01 to 1.13 mg·kg^−1^. Finally, petunidin-3-glucoside was the only anthocyanin found in samples H-5 and H-6, with values of 0.06 and 0.16 mg·kg^−1^, respectively. An important finding of this study was the major presence of phenolic compounds (mainly vanillic acid, caffeic acid, and quercetin) in *Coriandrum sativum* L. honey sample (H-13). Further studies are needed to characterize the phenolic profile and antioxidant capacity of *Coriandrum sativum* L. honeys of different origins. Regarding non-phenolic acids, only *trans*-cinnamic acid was quantified in honey samples and the highest concentration was found in *Castanea sativa* honey H-1 (0.59 mg·kg^−1^), the lowest in H-10 and H-14 (0.03 mg·kg^−1^), while it is not present in sample H-9.

### 2.4. Volatile Compounds by GC-MS

A total of 45 volatile compounds have been identified in honey samples.

The identified volatile compounds belonged to different chemical classes: ketones, alcohols, aldehydes, furanones, esters, pyrazines, furans, acids, and terpenes.

The relative abundance of volatiles in honey samples, their retention indexes (RIs), and their relative area percentage are shown in Table S2.

The main volatiles in *Castanea sativa* honeys (H-1, H-8) were benzyl alcohol (16.90% in H-1) and benzaldehyde (16.49% and 15.10%, respectively). Moreover, acetophenone, phenylethyl alcohol, cinnamaldehyde, furfural, hexanoic, octanoic, and nonanoic acids were present in both *Castanea sativa* honeys at lower proportions. On the contrary, butanoic acid, 3-methyl, butanoic acid, 2-methyl and butanoic acid, 3-hexenyl ester were detected only in H-1, while 3-methyl-3-buten-1-ol, linalool oxide, benzeneacetaldehyde, 3(2H)-furanone, dihydro-2-methyl, and 2-acetylfuran were detected only in H-8.

The main volatiles in *Robinia pseudoacacia* honeys (H-2, H-4, H-14) were benzaldehyde (43.44% and 28.99% in H-2 and H-4, respectively) and phenylethyl alcohol (55.87% in H-14). Linalool oxide, linalool, benzeneacetaldehyde, nonanal, pentanoic acid, 3-methyl, butanoic acid, 3-hexenyl ester, furfural, and nonanoic acid were present in *Robinia pseudoacacia* honey at lower percentages. As multiflower honeys are the product of different species of flowers, it is difficult to characterize the volatile composition and the aroma. In this study, multifloral honeys (H-3, H-5, H-7, H-9, H-12, H-15, H-16, H-17, H-18) were characterized mainly by furfural, nonanoic acid, benzeneacetaldehyde, furfural, 1,5,7-octatrien-3-ol,3,7-dimethyl, and nonanal. Linden honey (H-6) was characterized mainly by nonanal (16.22%), *p*-cymen-8-ol (12.79%), benzyl alcohol (11.31%), and, to a lesser extent, phenylethyl alcohol, terpinen-4-ol, benzaldehyde, furfural, and thymol. 2,6,6,-Trimethyl-2-cyclohexene-1,4-dione was the most abundant compound in *Arbutus unedo* L. honey (12.02%), followed by 2,3-butanediol, phenylethyl alcohol, benzeneacetaldehyde, benzaldehyde, nonanal, decanal, furfural, 2-acetylfuran, and thymol. Linalool oxide (16.70%) resulted in being the most abundant compound in *Helianthus annuus* honey, followed by nonanoic acid (12.80%), benzeneacetaldehyde (11.81%), and benzaldehyde (8.65%). In *Coriandrum sativum* L. honey the most abundant compounds were nonanal (36.06%), linalool (7.15%), and lilac aldehyde A (8.50%).

### 2.5. Statistical Analysis

**Figure 3:**
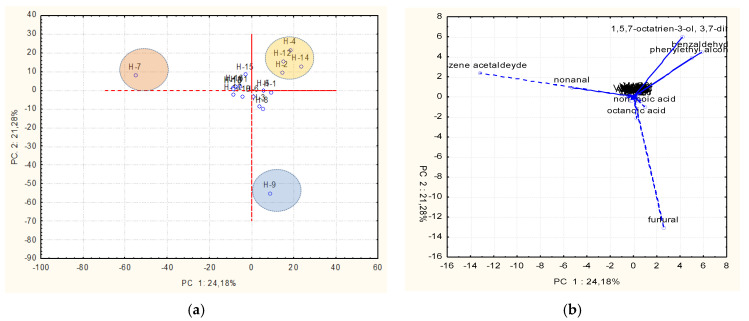
(**a**,**b**) Principal component analysis (PCA) of the volatile profiles of honey samples.

PCA was applied to study the possible correlation between the volatile profile and the botanical origin of the analyzed honey samples. The dataset revealed 45.46% of the total variance, distributed across two principal components (Figure 3). The PCA score plot (Figure 3a) and loading plot (Figure 3b) highlighted three distinct clusters, which were characterized by the presence of specific volatile organic compounds (VOCs) that serve as markers for differentiation among the samples.

The first cluster, dominated by benzene acetaldehyde, uniquely identified H-7 (multiflower honey), suggesting its role as a potential biomarker for this type. The second cluster corresponded to H-9 (blossom honey), characterized by furfural, a compound associated with Maillard reaction products or specific floral sources. The third cluster grouped H-2, H-4, H-12, and H-14 (*Robinia pseudoacacia* and multiflower honeys), characterized by 1,5,7-octatrien-3-ol, 3,7-dimethyl, benzaldehyde, and phenylethyl alcohol, which are compounds linked to aromatic and floral notes.

These results demonstrate the ability of PCA to differentiate honey types based on specific volatile organic compounds. Benzene acetaldehyde, furfural, and other key VOCs act as discriminant markers, reflecting the influence of nectar source, environmental factors, or processing conditions. Moreover, furfural is considered a quality marker of honey; in fact, it can be formed during the manufacturing and the preservation processes.

## 3. Discussion

TPC values were in very good accordance with those reported previously by Silici et al., 2010 [25], who reported a mean TPC of 866.7 mg GAE/kg in honey samples. The values are also comparable with previous results reporting a TPC of 231 to 1580 mg/kg of honey [11]. *Castanea sativa* honey from Portugal or Croatia showed lower TPC (487–1134 and 129.2–212.7 mg GAE/kg, respectively) than our *Castanea sativa* sample (848.36 mg GAE/kg) [26,27]. Similarly, *Helianthus annuus* and linden honey showed higher results than those reported by the literature [28,29]. These results highlighted the great influence of the botanical origin on honey composition. In fact, polyphenols content between samples of the same origin, even in honey of the same country, is different. Additionally, it is important to note that, although the Folin−Ciocalteu assay is commonly used to determine TPC in food extracts, it is not specific to phenolic compounds. Other substances present in honey, such as reducing sugars and amino acids, can also reduce the Folin−Ciocalteu reagent. Therefore, the measured TPC values might be higher than the actual phenolic content and for that we have analyzed phenolic profile using LC-MS/MS. Moreover, it must be taken into account that HPLC-MS/MS analysis allowed to quantify only 37 polyphenolic compounds. Some samples with similar TPC did not exhibit similar antioxidant capacities (as in H-15, 16, and 17, and H-14 and 18). This indicates that while phenols are a major class of natural antioxidants, the overall antioxidant capacity of each sample is due to the combined activity of various non-phenolic compounds. These include proteins, amino acids, peptide inhibitors of oxidative enzymes, enzymes like catalase and glucose oxidase, and organic acids such as gluconic, citric, and malic acids, which can chelate metals and enhance the action of other antioxidants like polyphenols [11]. The complex composition of honey suggests that interactions and possible synergies among different antioxidant compounds also significantly contribute to its overall antioxidant capacity. The colour of honey can range from a nearly colourless white, yellow, amber to a dark red [24]. Changes in the colour are related to the presence of pigments, and they depend on botanical origin, the composition of nectar, the process of acquisition, intervention of beekeepers, temperature, and storage time and conditions (use of old panels, contact with metals, light) [30]. The colour is the principal physical property that drives the consumer’s acceptability, and it can be crucial at the moment of purchase [31]. In fact, Delmoro et al. (2010) reported that consumers in North America are more attracted by lighter honeys with a more delicate flavour, while in Europe, consumers prefer darker honeys with a more intense flavour [32]. The colour of honey is also linked to the antioxidant capacity; in fact, the darker the colour, the greater the antioxidant action, which can be linked to the presence of anthocyanin and flavonic groups [33]. Moreover, several studies had positively correlated the colour of honey with the content of polyphenols, especially with flavonoid content [31,34,35,36,37]. In this study these results are confirmed; in fact, honey samples with high TPC values had a very dark colour (such as *Castanea sativa*, *Arbutus unedo* L. honey samples), while those with a low TPC value resulted in being very light (*Robinia pseudoacacia* honey samples). Polyphenols are a heterogeneous class of chemical compounds that can be divided into flavonoids (flavanols, flavones, flavanols, flavanones, anthocyanidin, chalcones, and isoflavones) and non-flavonoids (phenolic acids). They are the products of the secondary metabolism of plants and their presence in honey is due to the mixing of bees’ body fluids with the nectars of flowers or secretions of plants which contain water, sugars, proteins, and phenolic compounds [38,39]. The phenolic composition of honey can differ among the species, as it depends on its floral origin and, for this reason, it can be used for classification and authentication. However, some phenolic compounds are common among different species of honey: kaempferol and syringic acid in *Robinia pseudoacacia*, *Arbutus unedo* L. and thyme honey; myricetin, quercetin, caffeic acid, chlorogenic acid, ferulic acid, gallic acid, and *p*-coumaric acid in *Robinia pseudoacacia* and thyme honey; *p*-hydroxybenzoic acid in thyme honey; vanillic acid in *Robinia pseudoacacia* honey [6]. Results of phenolic acids were different from those found by Nascimento et al., 2018, who reported gallic acid as the most abundant phenolic compound found in Brazilian honeys and *p*-coumaric acid in samples of honey of Japanese grape, which confirms the view that the phenolic profile in honey is dependent on its botanical origin [40]. On the contrary, flavonoids composition was in good accordance with Combarros-Fuertes et al., 2019 [11], who reported quercetin, kaempferol, and isorhamnetin as major flavonoids in different honey samples. It is important to consider that the health benefits of polyphenolic compounds in honey depend on their bioavailability, absorption, and metabolism [6]. However, even if high molecular weight polyphenols are present in traces in the systemic circulation, they are responsible for the modulation of gut microbiota composition and activity or for the formation of metabolites which are more absorbed by the microbiota by deglycosylation, dihydroxylation, decarboxylation, hydrogenation, sulfation, glucuronidation, and C ring cleavage of the benzo-γ-pyrone system [41]. The presence of flavonoids like quercetin, isorhamnetin, kaempferol glycoside, and myricetin in the studied honey samples highlights the significance of including honey in a balanced diet, as it can help protect health against harmful effects. Foods rich in phenolic compounds, such as plants and their derivatives, including honey, are highly valued and crucial parts of diets due to their numerous antioxidant-related health benefits. Volatile composition was studied in honey samples. *Castanea sativa* honey is characterized by a bitter, sweet, burnt caramel and woody flavour [42,43]. These results were reported in the literature, with benzaldehyde as the predominant volatile in *Castanea sativa* honeys from eight different countries (Croatia, France, Italy, Germany, Greece, Portugal, Spain, and Turkey) [44]. Benzyl alcohol, instead, was found as one of the predominant volatiles in honeys from Croatia, France, and Italy [45,46,47]. Moreover, furfural was found in honeys from Croatia, France, Germany, and Greece [42,45,48]. Nonanoic acid and linalool oxide were found in *Castanea sativa* honey from Italy [49], while 2-acetylfuran in honeys from France and Germany [42]. *Robinia pseudoacacia* honey was characterized by a sweet, beeswax, and sourish flavour [45]. Most of these compounds were reported to be present in *Robinia pseudoacacia* honey: benzaldehyde and furfural in honey from Austria, Czech Republic, France, Germany, Italy, Morocco, Poland, Romania, Spain; linalool oxide in honeys from Austria, Morocco, Romania, Slovakia, Spain; benzeneacetaldehyde in honey from Morocco [44]. Linden honey has a sweet, bitter, medicinal, floral, woody, and hay-like taste [45]. These results were in accordance with those reported by Machado et al. [44], in which linden honeys from China, Croatia, Czech Republic, France, Germany, Netherlands, Poland, Romania, and Slovakia were rich in furfural, terpinene-4-ol, and benzyl alcohol.

*Arbutus unedo* L. honey has a peculiar fragrance, characterized by a pleasant bitter aftertaste; however, its volatile composition is not fully investigated [50,51]. Some of these compounds (especially nonanal) were reported also in other studies on *Arbutus unedo* L. honey’s volatiles [48]. *Helianthus annuus* honey, mainly produced in eastern and southern Europe, has a fruity, warm, and vegetal aroma [52]. These results were confirmed by the literature as benzaldehyde and benzeneacetaldehyde resulted in being the predominant compounds in *Helianthus annuus* honey from France, Italy, and Turkey [42,53]. *Coriandrum sativum* L. honey resulted in having a strong olfactory fragrance of citrus, and, to a lesser extent, of fatty and unpleasant soap odours. Aldehydes resulted in being the most important volatiles in *Coriandrum sativum* L., along with linalool, confirming the results obtained for *Coriandrum sativum* L. honey [54].

## 4. Materials and Methods

### 4.1. Chemical and Reagents

Cyanidin-3-glucoside chloride, delphinidin-3,5-diglucoside chloride, delphinidin-3-galactoside chloride, petunidin-3-glucoside chloride, malvidin-3-galactoside chloride, quercetin-3-glucoside, and kaempferol-3-glucoside were purchased from PhytoLab (Vestenbergsgreuth, Germany). The remaining 29 analytical standards of the 37 analytes were supplied by Sigma-Aldrich (Milan, Italy). Individual stock solutions of each analyte, at a concentration of 1000 mg·L^−1^, were prepared by dissolving pure standards in HPLC-grade methanol. All solvents and solutions were filtered through a 0.2 μm polyamide filter from Sartorius Stedim (Goettingen, Germany). Before HPLC analysis, all samples were filtered with Phenex™ RC 4 mm 0.2 μm syringeless filter, Phenomenex (Castel Maggiore, BO, Italy). Standard stock solutions at different concentrations (0.001–0.005–0.01–0.05–0.1–0.5–1 mg∙kg^−1^) were prepared to build calibration curves to calculate the concentrations of samples (Table S3).

### 4.2. Honey Samples

Honey samples were purchased from an Italian market, and they were produced by different Italian companies such as Fior di Loto, Melizia Bio, Wild Flowers, Mariangela Prunotto, Agrisicilia, Adi Apicoltura, Ambrosoli, Apicoltura Bianco, and Menz&Gasser. Eighteen honey samples were used for all analysis: nine samples of unifloral honeys (two *Castanea sativa*, linden, *Arbutus unedo* L., *Helianthus annuus*, *Coriandrum sativum* L., and three *Robinia pseudoacacia* samples) and nine samples of a multifloral honey were analyzed (Table 3 and Figures S2 and S3).

In the label of five of them (H-2, H-10, H-15, H-16, and H-17), one or more foreigner geographical origin was indicated.

### 4.3. Spectrophotometric Analysis

Honey samples were put in a falcon tube with methanol (1:3 ratio), then vortexed and used for the spectrophotometric analysis using Agilent Cary 8454 UV–Vis spectrophotometer (Agilent Technologies, Woburn, MA, USA). Measurements were performed in triplicate.

#### 4.3.1. Total Phenolic Content (TPC)

TPC was determined using the method described by Mustafa et al. (2016), with some modifications [55]. Briefly, 0.5 mL of the methanolic extract was mixed with 2.5 mL of diluted Folin–Ciocalteu solution and 7 mL of Na_2_CO_3_ (7.5% aqueous solution), then left for 2 h in dark at 25 °C. The absorption was measured spectrophotometrically at 700 nm. A gallic acid calibration curve was used to determine the TPC of samples. Results were expressed as mg of gallic acid equivalents (GAE) per kg sample (mg GAE/kg).

#### 4.3.2. Total Flavonoid Content (TFC)

TFC was determined by the spectrophotometric method described by Laurita et al. [56]. Briefly, 0.5 mL of extract was mixed with 0.15 mL of 0.5 M NaNO_2_, 3.2 mL of 30% methanol (*v*/*v*), and 0.15 mL of 0.3 M AlCl_3_. Then, 1 mL of NaOH (1 M) was added after 5 min. The reaction mixture was mixed well, and the absorbance was determined using UV spectrophotometer against a blank reagent at 506 nm. The standard calibration curve for TFC was made using rutin standard solutions and the TFC was calculated as mg of rutin equivalents (RE) per kg sample (mg RE/kg).

#### 4.3.3. Antioxidant Activity (AOA)

The antioxidant activity was determined spectrophotometrically using the DPPH method, described by Mustafa et al. [57]. Briefly, 0.5 mL of extract solution was mixed with 4.5 mL of ethanolic solution of DPPH (0.1 mM). After 30 min of incubation in the dark at room temperature, the DPPH disappearance was measured spectrophotometrically at 517 nm. Trolox was used as the reference antioxidant and the results were expressed as mg trolox equivalent (TE) per kg sample (mg TE/kg).

### 4.4. Colour Determination

Honey samples were heated to 50 °C to dissolve the sugar crystals and the colour was determined by spectrophotometric measurement of the absorbance of a 50% (*w*/*v*) honey solution at 635 nm. The samples were classified according to the Pfund scale after conversion of absorbance values: mm [58].

Pfund = −38.70 + 371.39 × Abs

### 4.5. Phenolic Profile Analysis Using LC-MS/MS

#### 4.5.1. Extraction of Phenolic Compounds

The extraction and purification of phenolic compounds from honey was performed according to the method described by Kenjerić et al. [59], with slight modifications. The honey sample (2.5 g) was mixed with 2.5 mL of 10% HCl solution (pH = 2), until it was totally fluid. Then, this solution was purified using solid-phase extraction (SPE) cartridge such as Strata C18-E (500 mg/5 mL). The phenolic compounds were retained in the column, while sugars and other polar compounds were eluted with the aqueous solvent. The column was preconditioned with methanol followed by 37% HCl solution (pH = 2). After that, 5 mL of sample was purified through the cartridges, then washed with 4 mL of deionised water and finally eluted with 2 mL of 90% *v*/*v* methanolic solution. Samples were then dried by nitrogen, and the residue was redissolved in 0.5 mL methanol.

#### 4.5.2. HPLC-MS/MS Analysis

Phenolic compounds in honey extracts have been analyzed using the method described by Mustafa et al. [57]. An Agilent 1290 Infinity series coupled with Triple Quadrupole 6420 from Agilent Technology (Santa Clara, CA, USA) equipped with an electrospray ionization (ESI) source operating in negative and positive ionization modes was used for the analysis. Phenolic compounds were separated on a Synergi Polar-RP C18 analytical column (250 mm × 4.6 mm, 4 µm) by Phenomenex (Chesire, UK), coupled with a pre-column Polar RP security guard cartridge (4 mm × 3 mm ID). The mobile phase was a mixture of (A) water and (B) methanol, both with formic acid 0.1%, at a flow rate of 0.8 mLmin^−1^ in gradient elution mode. The elution programme used for the separation was 0–1 min, isocratic condition, 20% B; 1–25 min, 20–85% B; 25–26 min, isocratic condition, 85% B; 26–32 min, 85–20% B. All solvents and solutions were filtered through a 0.2 μm polyamide filter from Sartorius Stedim (Goettingen, Germany). The injection volume was 2 μL. The temperature of the column was 30 °C, and the temperature of the drying gas in the ionization source was 350 °C. The gas flow was 12 L/min, the nebulizer pressure was 55 psi, and the capillary voltage was 4000 V.

Detection was performed in the dynamic-multiple reaction monitoring (dynamic-MRM) mode, and the dynamic-MRM peak areas were integrated for quantification. The most abundant product ion was used for quantitation, and the others for qualification. The specific time window for each compound (Δ retention time) was set at 2 min. The selected ion transitions and the mass spectrometer parameters for the analyzed compounds are reported in Table S4. A chromatogram was reported in Figure S4.

### 4.6. Volatile Profile Analysis Using GC-MS

#### 4.6.1. Extraction of Volatile Compounds

The extraction was performed using a HS-SPME method. Briefly, 5 g of sample were placed in a 20 mL vial which was tightly clapped with a PTFE/silicon septum. The sample was incubated for 40 min at 50 °C and then extracted for 15 min under stirring with 5 s of on-time and 2 s of off-time. The extraction was performed by a divinylbenzene/carbon-wide range/polydimethylsiloxane (DVB/C-WR/PDMS) 80 μm fibre, after a preconditioning of 15 min at 250 °C. After the extraction, the fibre was inserted into the injector port at a speed of 100 mm s^−1^ and a penetration depth of 40 mm at 250 °C for 3 min for the desorption.

#### 4.6.2. GC-MS Analysis

Volatile compounds were analyzed through a GC-MS, composed by an Agilent 8890 GC equipped with a PAL RTC 120 autosampler and coupled to an Agilent 5977B MSD quadrupole detector with an electron ionization (EI) source (Santa Clara, CA, USA).

The injector temperature was set at 250 °C, and the liner used was recommended for SPME injection, namely, Inlet liner, Ultra Inert, splitless, straight, 0.75 mm id, from Agilent. The gas carrier was helium at flow rate of 1 mL·min^−1^.

The separation of volatiles was performed with a HP-5MS UI column (30 m, 250 μm id, 0.25 μm film thickness). The oven temperature programme was set as follows: initial temperature at 35 °C, then increased to 60 °C at a rate of 7 °C/min for 1 min, then increased to 80 °C at 1.5 °C/min, then to 110 °C at 1.5 °C/min, and finally 280 °C at 10 °C/min and held for 1 min [60]. EI and transfer line temperatures were set at 250 and 280 °C, respectively. The acquisition was performed in SCAN mode (35–450 m/z) and peak identification was performed by comparing the mass spectrum and experimental linear retention index (RI) with data from the NIST library (US National Institute of Standards and Technology) and those reported in the literature. The data results were managed by the MSD ChemStation software (Agilent, version G1701DA D.01.00). A chromatogram was reported in Figure S4.

### 4.7. Statistical Analysis

Data on volatile compounds were examined by principal component analysis (PCA) using Statistica v.7.1 (Stat Soft Italia, Vigonza, Italy). Two covariance data matrices were created: one included 37 variables and 18 samples (polyphenols and different honey samples) and the other one 52 variables and 18 samples (volatile compounds and different honey samples). The data were analyzed using principal component analysis (PCA) with STATISTICA 7.1 (Stat Soft Italia srl, 2005, www.statsoft.it (accessed on 1 January 2025)).

## 5. Conclusions

This study highlighted the high potential of honey in terms of polyphenolic and volatile profiles and antioxidant activity. In particular, the high content of phenolic compounds was related to a high antioxidant activity, which is desirable to maintain a good health. This was strictly connected to the colour of honey; in fact, darker honey generally showed a higher antioxidant activity due to the presence of anthocyanins and flavonoids. Finally, honeys had a rich volatile profile, characterized by different compounds among the samples, confirming the high variability among honeys of different origins. However, PCA showed that samples H-7 and H-9 can be characterized by benzene acetaldehyde and furfural, respectively, which are not present in other samples. In addition, samples H-2, H-4, H-12, and H-14 also differed from other samples, due to the presence of 1,5,7-octatrien-3-ol,3,7-dimethyl, benzaldehyde, and phenylethyl alcohol.

This study has increased our knowledge about honey in terms of chemical composition (polyphenols and volatiles), antioxidant, and colour properties laying the foundations for further research into possible applications in the industrial and food fields.

## Figures and Tables

**Table 1:** TPC, TFC, and DPPH values obtained from spectrophotometric analysis. All data are expressed as mg·kg^−1^ with standard deviation.

	TPC (mg GAE/kg)	TFC (mg RE/kg)	DPPH (mg TE/kg)
H-1 *Castanea sativa*	868.41 ± 152.6	195.20 ± 17.0	635.54 ± 49.1
H-2 *Robinia pseudoacacia*	232.40 ± 48.9	56.15 ± 5.6	37.01 ± 20.3
H-3 Multiflower	615.47 ± 26.9	298.59 ± 51.1	422.36 ± 73.5
H-4 *Robinia pseudoacacia*	406.03 ± 155.2	57.81 ± 14.7	36.48 ± 27
H-5 Multiflower	635.05 ± 164.4	165.80 ± 40.3	446.34 ± 28.1
H-6 *Castanea sativa*	414.92 ± 99.3	108.88 ± 12.9	257.27 ± 249.2
H-7 Multiflower	597.43 ± 151.7	181.60 ± 34.3	267.78 ± 133.2
H-8 *Castanea sativa*	848.36 ± 226	227.88 ± 41.6	670.92 ± 13.3
H-9 Blossom honey	246.90 ± 75.9	36.15 ± 29.6	94.65 ± 14.3
H-10 *Arbutus unedo* L.	761.11 ± 196.5	292.44 ± 69.6	583.75 ± 98.2
H-11 *Helianthus annuus*	549.56 ± 146.8	307.90 ± 70.5	244.06 ± 155.6
H-12 Multiflower	505.59 ± 143.9	236.16 ± 82.2	460.96 ± 243.2
H-13 *Coriandrum sativum* L.	554.84 ± 83.4	242.69 ± 110.1	360.33 ± 69.3
H-14 *Robinia pseudoacacia*	379.64 ± 69.4	50.35 ± 29.9	19.74 ± 6.6
H-15 Blossom honey	176.18 ± 8.3	203.79 ± 57.7	234.37 ± 54.1
H-16 Blossom honey	190.08 ± 22.6	127.40 ± 53.7	23.53 ± 90.9
H-17 Multiflower	216.99 ± 18.5	210.31 ± 23.7	23.54 ± 64.1
H-18 Multiflower	401.16 ± 24.9	68.34 ± 7.9	17.14 ± 281.4

**Table 2:** Colour determination in honey samples with Pfund values (mm) and colour attributes.

Name	Scale Pfund (mm)	Colour
-1 *Castanea sativa*	90 ± 2.8	Amber
-2 *Robinia pseudoacacia*	32.1 ± 6.7	White to very light amber
-3 Multiflower	203.6 ± 4.8	Dark amber
-4 *Robinia pseudoacacia*	31.1 ± 4.5	White to very light amber
-5 Multiflower	135.9 ± 4.9	Dark amber
-6 *Castanea sativa*	82.5 ± 11.3	Amber
-7 Multiflower	92.4 ± 1.1	Amber
-8 *Castanea sativa*	141.3 ± 0.2	Dark amber
-9 Blossom honey	10 ± 2.9	Light white
-10 *Arbutus unedo* L.	204.7 ± 12.1	Dark amber
-11 *Helianthus annuus*	191.7 ± 17.6	Dark amber
-12 Multiflower	152.2 ± 10.3	Dark amber
-13 *Coriandrum sativum* L.	169.9 ± 4.7	Dark amber
-14 *Robinia pseudoacacia*	39.3 ± 5	Very light amber
-15 Blossom honey	171.32 ± 1.3	Dark amber
-16 Blossom honey	151.08 ± 0.5	Dark amber
-17 Multiflower	151.70 ± 27	Dark amber
-18 Multiflower	141.18 ± 5.6	Dark amber

**Table 3:** Botanical origin, glucose content, quality brand, geographical origin, and year of production of Italian honey samples.

Sample Name	Botanical Origin	Glucose Content (mg kg^−1^)	Brand	Geographical Origin	Year of Production
H-1	*Castanea sativa*	80 g/100 g	Fior di loto	Italy	2023
H-2	*Robinia pseudoacacia*	83 g/100 g	Mielizia Bio	Italy, Hungary	2023
H-3	Multifloral	-	Wild Flowers	Italy	2023
H-4	*Robinia pseudoacacia*	80 g/100 g	Mariangela Prunotto	Italy	2023
H-5	Multifloral	80 g/100 g	Mariangela Prunotto	Italy	2023
H-6	Linden	80 g/100 g	Fior di loto	Italy	2023
H-7	Multifloral	80.3 g/100 g	Agrisicilia	Italy	2023
H-8	*Castanea sativa*	80.3 g/100 g	Agrisicilia	Italy	2023
H-9	Multifloral	-	Menz&Gasser	Italy	2023
H-10	*Arbutus unedo* L.	68.20 g/100 g	-	Greece	2023
H-11	*Helianthus annuus*	82 g/100 g	Apicoltura Bianco	Italy	2023
H-12	Multifloral	-	Azienda Luca Bianchi	Italy	2022
H-13	*Coriandrum sativum* L.	-	Azienda Luca Bianchi	Italy	2022
H-14	*Robinia pseudoacacia*	-	Azienda Luca Bianchi	Italy	2022
H-15	Multifloral	80.6 g/100	Ambrosoli	Italy, Argentina, Hungary, Moldova	2023
H-16	Multifloral	80.6 g/100	Ambrosoli	Italy, Argentina, Hungary, Moldova	2023
H-17	Multifloral	80.6 g/100	Ambrosoli	Italy, Argentina, Hungary, Moldova	2023
H-18	Multifloral	80 g/100	Adi Apicoltura	Italy	2023

## Data Availability

The original contributions presented in this study are included in the article/Supplementary Materials. Further inquiries can be directed to the corresponding author(s).

## Supplementary Materials

The following supporting information can be downloaded at: https://www.mdpi.com/article/10.3390/plants14193109/s1, Figure S1: Colour of four different types of honey ranging from white to very dark amber. (A) H-2 (B) H-9 (C) H-5 (D) H-10; Figure S2: (A) miele di castagno H-1; (B) miele d’acacia H-2; (C) miele millefiori H-3; (D) miele d’acacia H-4; (E) miele millefiori H-5; (F) miele di tiglio H-6; (G) miele millefiori H-7; (H) miele di castagno H-8; (I) miele di fiori H-9; (L) miele di fragola H-10; (M) miele di girasole H-11; (N) miele millefiori dell’azienda H-12; (O) miele al coriandolo dell’azienda H-13; (P) miele d’acacia dell’azienda H-14; (Q) miele di fiori H-15; (R) miele di fiori H-16; (S) miele millefiori H-17; (T) miele millefiori H-18; Figure S3: Geographical origin of honey samples; Figure S4: HPLC-MS/MS and GC-MS chromatograms of a sample of honey; Table S1: Concentration (mg·Kg^−1^) of polyphenols in honey samples; Table S2: Volatile compounds (%) in honey samples; Table S3: Calibration curves of the 37 marker compounds parameters; Table S4: HPLC–MS/MS acquisition parameters (dynamic-MRM mode) used for the analysis of the 37 marker compounds.
