# Supplementary material for: Analysis of the Phenolic Compounds, Volatile Profile, and Evaluation of the Antioxidant Activity of 18 Different Varieties of Honey from the Italian Market
Source: Plants (Basel). 2025 Oct 9;14(19):3109. doi: 10.3390/plants14193109 (PMC12526456; doi:10.3390/plants14193109)
Supplement: Supplementary file 1 [file plants-14-03109-s001.zip › plants-3803217-supplementary.pdf]

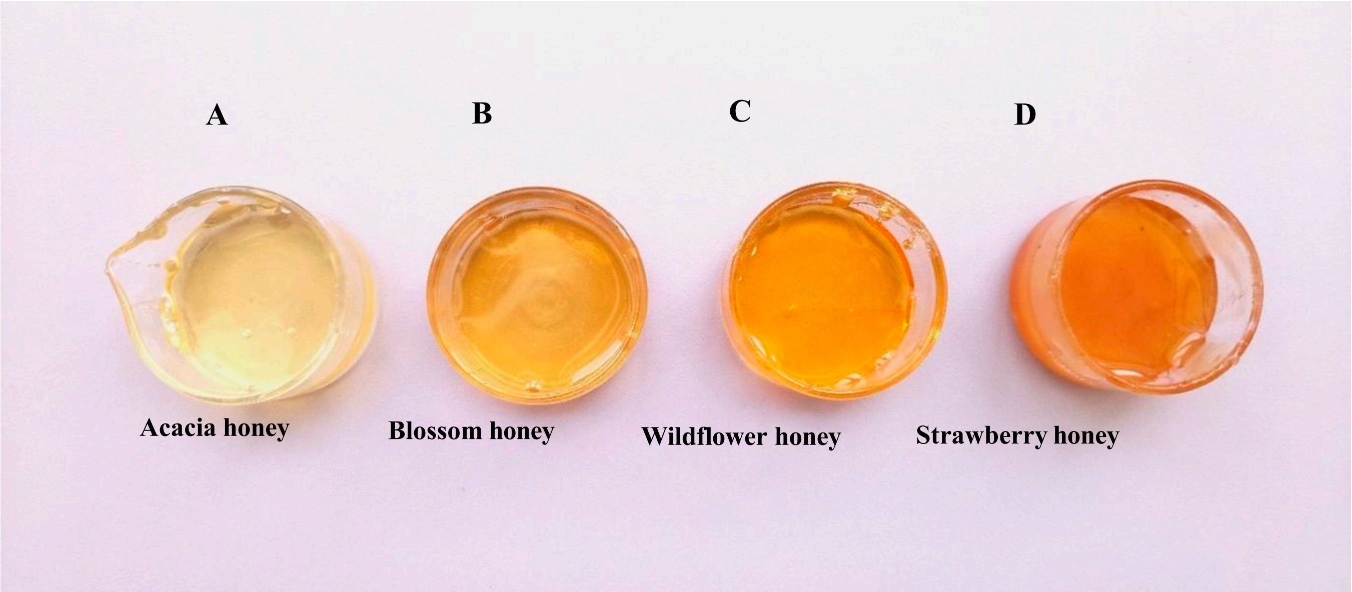

Figure S1: Colour of four different types of honey ranging from white to very dark amber. A) H-2 B) H-9 C) H-5 D) H-10.

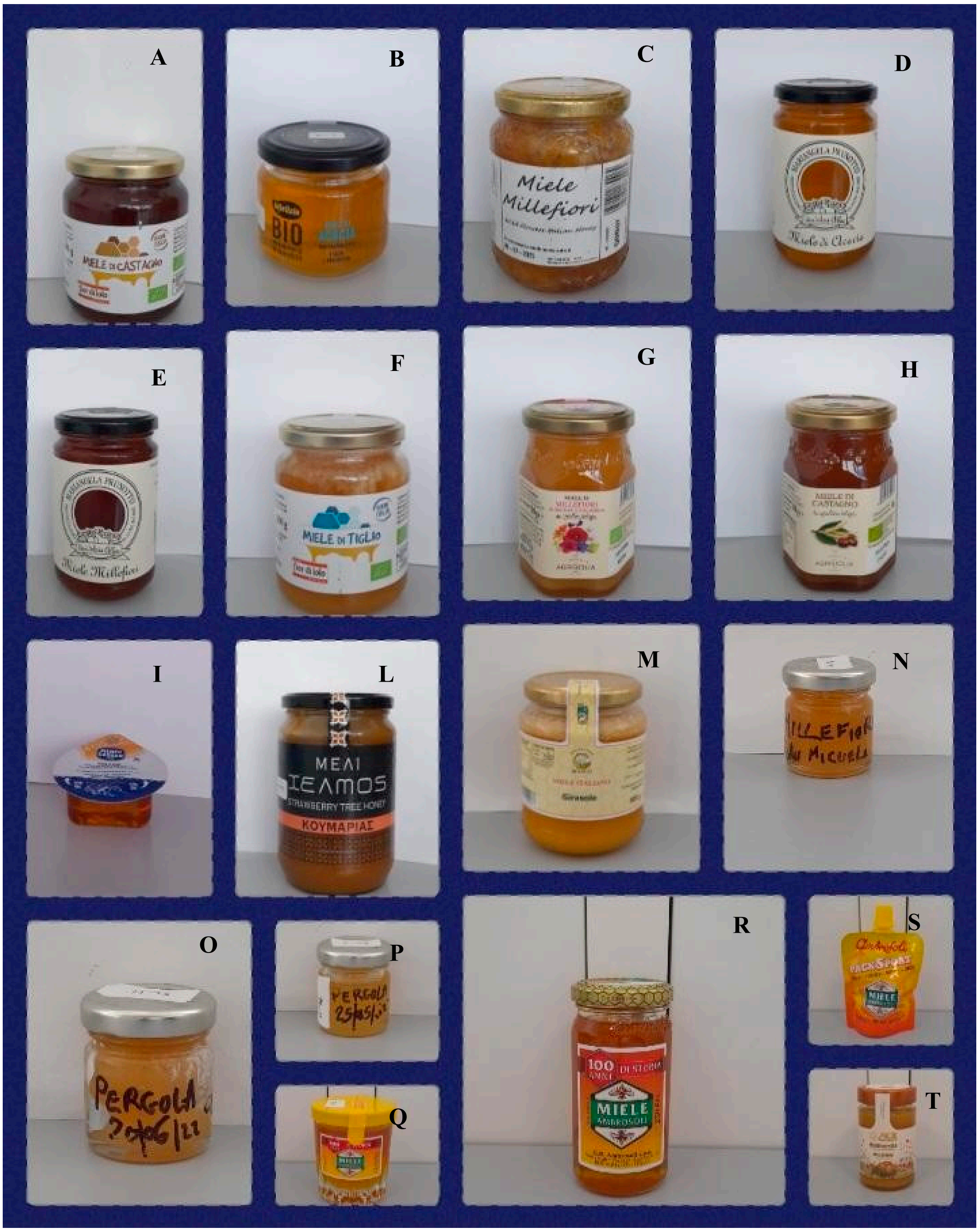

Figure S2: **A)** miele di castagno H-1; **B)** miele d'acacia H-2; **C)** miele millefiori H-3; **D)** miele d'acacia H-4; **E)** miele millefiori H-5; **F)** miele di tiglio H-6; **G)** miele millefiori H-7; **H)** miele di castagno H-8; **I)** miele di fiori H-9; **L)** miele di fragola H-10; **M)** miele di girasole H-11; **N)** miele millefiori dell'azienda H-12; **O)** miele al coriandolo dell'azienda H-13; **P)** miele d'acacia dell'azienda H-14; **Q)** miele di fiori H-15; **R)** miele di fiori H-16; **S)** miele millefiori H-17; **T)** miele millefiori H-18.

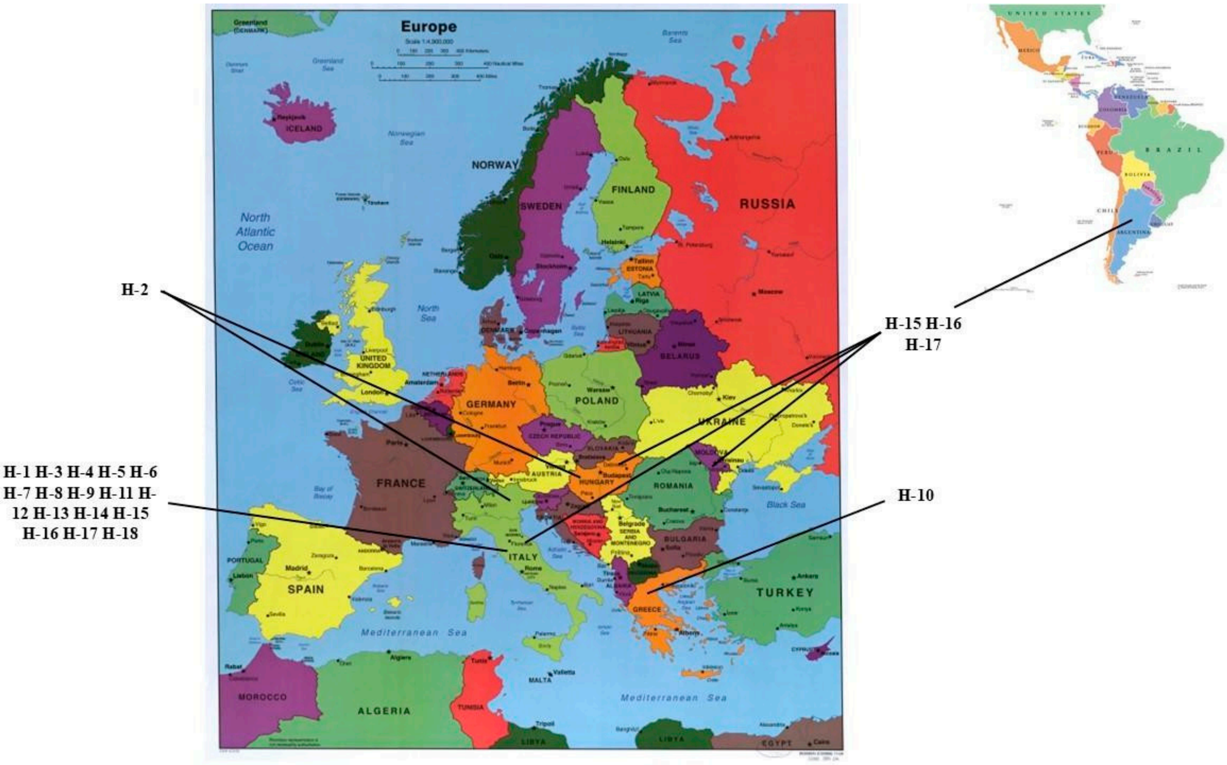

Figure S3: Geographical origin of honey samples.

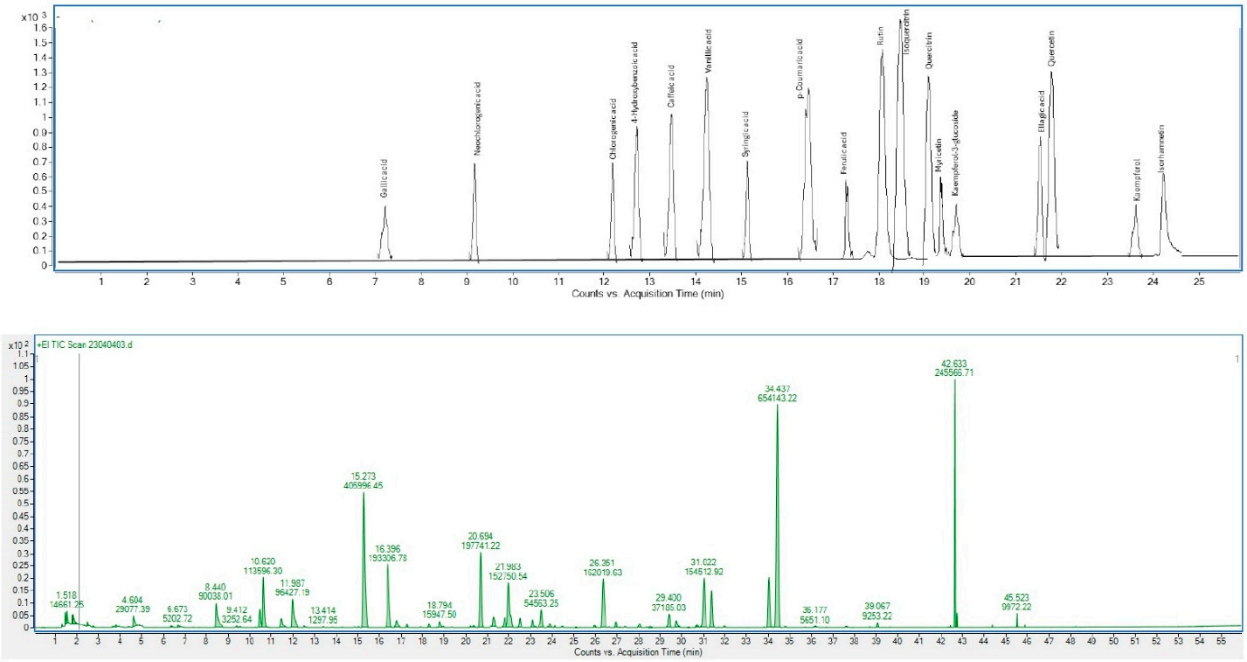

Figure S4: HPLC-MS/MS and GC-MS chromatograms of a sample of honey.

Table S1. Calibration curves of the 37 marker compounds parameters.

No.	Compounds	Conc. range (mg/L)	R^2 [a]	LOD (mg/L)[b]	LOQ (mg/L)[c]
1	Gallic acid	0.005–	0.9956	0.003	0.009

No.	Compounds	Conc. range (mg/L)	R^2 [a]	LOD (mg/L)[b]	LOQ (mg/L)[c]
		10			
2	Neochlorogenic acid	0.005–10	0.9984	0.002	0.006
3	Delphinidin-3-galactoside	0.005–10	0.9988	0.0016	0.005
4	(+)-Catechin	0.005–10	0.9977	0.0026	0.008
5	Procyanidin B2	0.005–10	0.9961	0.0033	0.01
6	Chlorogenic acid	0.005–10	0.9993	0.0033	0.01
7	*p*-Hydroxybenzoic acid	0.005–10	0.9980	0.0027	0.0083
8	(-)-Epicatechin	0.005–10	0.9977	0.0025	0.0075
9	Cyanidin-3-glucoside	0.005–10	0.9967	0.0023	0.0070
10	Petunidin-3-glucoside	0.005–10	0.9970	0.001	0.0029
11	3-Hydroxy benzoic acid	0.005–10	0.9992	0.0024	0.0072
12	Caffeic acid	0.005–10	0.9985	0.0015	0.0045
13	Vanillic acid	0.005–10	0.9943	0.0033	0.01
14	Pelargonidin-3-glucoside	0.005–10	0.9979	0.0009	0.0027
15	Pelagonidin-3-rutinoside	0.005–10	0.9976	0.0012	0.0036
16	Malvidin-3-galactoside	0.005–10	0.9954	0.001	0.0030
17	Syringic acid	0.005–10	0.9997	0.0032	0.01
18	Procyanidin A2	0.005–10	0.9998	0.0033	0.0099
19	*P*-Coumaric acid	0.005–10	0.9990	0.0031	0.0093
20	Ferulic acid	0.005–10	0.9959	0.0013	0.0039
21	3,5-Dicaffeoylquinic acid	0.005–10	0.9999	0.0037	0.0111
22	Rutin	0.005–	0.9985	0.003	0.009

No.	Compounds	Conc. range (mg/L)	$R^{2\,a}$	LOD (mg/L)[b]	LOQ (mg/L)[c]
		10			
23	Hyperoside	0.005–10	0.9972	0.002	0.006
24	Isoquercitrin	0.005–10	0.9999	0.0025	0.008
25	Delphinidin-3,5-diglucoside	0.005–10	0.9998	0.0019	0.006
26	Phloridzin	0.005–10	0.9995	0.0022	0.0066
27	Quercitrin	0.005–10	0.9990	0.003	0.009
28	Myricetin	0.005–10	0.9992	0.0026	0.008
29	Naringin	0.005–10	0.9970	0.0017	0.0051
30	Kaempferol-3-glucoside	0.005–10	0.9950	0.0016	0.005
31	Hesperidin	0.005–10	0.9994	0.0017	0.0051
32	Ellagic acid	0.005–10	0.9999	0.0033	0.01
33	Quercetin	0.005–10	0.9998	0.0015	0.0045
34	Phloretin	0.005–10	0.9951	0.0006	0.002
35	Kaempferol	0.005–10	0.9989	0.0026	0.008
36	Isorhamnetin	0.005–10	0.9987	0.0004	0.0012

[a]R^2: Coefficient of determination

Table S2. HPLC–MS/MS acquisition parameters (dynamic-MRM mode) used for the analysis of the 37 marker compounds.

No.	Compounds	Precursor ion, *m*/*z*	Product ion, *m*/*z*	Fragm-entor, V	Collision energy, V	Polarity	Retention time (Rt, min)
1	Gallic acid	169	125.2*	97	12	Negative	6.96
2	Neochlorogenic acid	353	191.2*, 179	82	12, 12	Negative	9.52
3	Delphinidin-3-galactoside	465.01	303*	121	20	Positive	11.36
4	(+)-Catechin	289	245.2*,109.2	131	8, 20	Negative	11.44
5	Procyanidin B2	576.99	576.99*, 321.2	160	0, 32	Negative	12.41
6	Chlorogenic acid	353	191.2*, 127.5	82	12, 20	Negative	12.42
7	*p*-Hydroxybenzoic acid	137	93.2*	92	16	Negative	12.86

8	(−)-Epicatechin	289	245.1*, 109.1	126	8, 20	Negative	13.03
9	Cyanidin-3-glucoside	449	287.3*, 255.6	121	20, 20	Positive	13.14
10	Petunidin-3-glucoside	479.01	317*, 302	121	20, 44	Positive	13.26
11	3-Hydroxybenzoic acid	137	93.2*	88	8	Negative	13.59
12	Caffeic acid	179	135.2*, 134.1	92	12, 24	Negative	13.65
13	Vanillic acid	167	152.4*, 108.1	88	12, 20	Negative	14.32
14	Pelargonidin-3-glucoside	433.01	271*, 121	116	24, 50	Positive	14.52
15	Pelagonidin-3-rutinoside	579.01	271*	145	32	Positive	14.56
16	Malvidin-3-galactoside	493.01	331*, 315.1	121	20, 50	Positive	14.64
17	Syringic acid	196.9	182.2*, 121.2	93	8, 12	Negative	15.28
18	Procyanidin A2	575	575*, 285	170	0, 20	Negative	16.18
19	*p*-Coumaric acid	163	119.2*, 93.2	83	12, 36	Negative	16.70
20	Ferulic acid	193	134.2*, 131.6	83	12, 8	Negative	17.10
21	3,5-Dicaffeoylquinic acid	514.9	353.1*, 191	117	8, 28	Negative	17.61
22	Rutin	609	300.2*, 271.2	170	32, 50	Negative	17.73
23	Hyperoside	465.01	303*, 61.1	97	8, 50	Positive	18.33
24	Isoquercitrin	463	271.2*, 300.2	155	44, 24	Negative	18.36
25	Delphinidin-3,5 diglucoside	462.9	300.1*	165	24	Negative	18.38
26	Phloridzin	435.39	273*, 167	155	8, 28	Negative	18.83
27	Quercitrin	446.99	300.2*, 301.2	160	24, 16	Negative	19.61
28	Myricetin	316.99	179.1*, 182	150	16, 24	Negative	19.61
29	Naringin	578.99	271.3*, 151.3	170	32, 44	Negative	19.62
30	Kaempferol-3-glucoside	447	284.2*, 255.2	170	24, 40	Negative	19.77
31	Hesperidin	611.01	303*, 334.8	112	20, 12	Positive	20.19
32	Ellagic acid	301	301*, 229	170	0, 24	Negative	21.41
33	*Trans*-Cinnamic acid	149	131.2*, 77.2	74	4, 36	Positive	21.44
34	Quercetin	300.99	151.2*, 179.2	145	16, 12	Negative	21.87
35	Phloretin	272.99	167*, 123	116	8, 20	Negative	22.30
36	Kaempferol	287.01	153*, 69.1	60	36, 50	Positive	23.84
37	Isorhamnetin	314.99	300.2*, 196.1	145	16, 4	Negative	24.57

*These product ions were used for quantification.

Table S3. Concentration ($mg \cdot Kg^{-1}$) of polyphenols in honey samples.

	Compounds	H-1	H-2	H-3	H-4	H-5	H-6	H-7	H-8	H-9	H-10	H-11	H-12	H-13	H-14	H-15	H-16	H-17	H-18
	**Phenolic acids TOTAL**	**57.67**	**51.43**	**91.98**	**88.75**	**70.88**	**38.40**	**38.69**	**48.63**	**18.64**	**24.60**	**70.48**	**149.42**	**230.05**	**33.15**	**85.46**	**102.61**	**125.19**	**113.93**
**1**	Gallic acid	0.04±0.0	0.13±0.0	0.22±0.0	0.20±0.0	0.07±0.0	0.03±0.0	0.21±0.0	0.16±0.0	0.04±0.0	1.06±0.0	0.06±0.0	0.18±0.0	0.24±0.1	0.06±0.0	0.12±0.0	0.12±0.0	0.11±0.0	0.18±0.0
**2**	Neochlorogenic acid	0.02±0.0	0.06±0.0	0.84±0.0	0.12±0.0	0.19±0.0	0.02±0.0	0.24±0.0	0.11±0.0	0.16±0.0	0.04±0.0	2.42±0.0	0.18±0.0	0.18±0.0	0.01±0.0	2.18±0.2	2.47±0.3	2.44±0.2	0.87±0.2
**3**	Chlorogenic acid	0.05±0.0	0.18±0.1	2.58±0.1	0.33±0.0	0.57±0.0	0.05±0.0	0.44±0.0	0.25±0.0	0.54±0.0	0.06±0.0	7.85±0.0	0.54±0.0	1.42±0.6	0.04±0.0	8.76±0.6	9.63±0.8	10.13±0.7	2.20±0.2
**4**	4-Hydroxybenzoic acid	3.44±1.9	7.97±2.9	21.38±1.3	11.75±0.7	10.88±1.5	4.94±0.3	8.40±0.2	8.23±0.9	4.71±0.2	9.35±0.0	15.11±0.0	10.17±0.2	14.43±5.9	3.86±0.0	13.08±0.8	15.92±1.7	17.81±0.8	12.25±0.3
**5**	3-Hydroxybenzoic acid	0.37±0.0	0.00±0.0	0.24±0.0	0.00±0.0	17.85±2.5	0.00±0.0	0.00±0.0	0.00±0.0	0.00±0.0	0.40±0.2	0.00±0.0	0.44±0.0	0.46±0.1	0.00±0.0	0.00±0.0	0.00±0.0	0.00±0.0	0.26±0.1
**6**	Caffeic acid	7.93±2.9	8.29±1.4	15.07±2.0	11.56±0.3	7.58±7.2	5.96±0.6	3.88±0.5	3.50±0.1	0.58±0.2	2.56±0.0	13.28±0.0	36.00±2.3	39.63±17.1	8.71±0.0	8.46±0.6	7.30±0.6	8.54±0.5	14.64±0.5
**7**	Vanillic acid	29.73±25.2	17.07±13.4	30.18±3.9	29.14±5.7	11.99±2.3	6.80±1.1	19.50±2.7	29.65±11.6	7.96±0.7	7.99±1.0	0.00±0.0	64.51±6.6	119.73±63.4	8.55±0.0	33.15±3.6	43.47±6.7	54.78±0.7	49.77±4.3
**8**	Syringic acid	0.20±0.1	0.46±0.4	1.68±0.2	0.98±0.0	0.61±0.3	0.06±0.0	0.41±0.1	0.36±0.1	0.95±0.0	0.27±0.0	5.07±0.0	4.55±0.1	7.71±4.9	3.53±0.0	0.91±0.1	1.13±0.2	1.42±0.0	1.45±0.1
**9**	*p*-Coumaric acid	9.67±3.9	9.22±3.3	8.70±0.6	17.52±0.8	12.08±1.9	13.95±1.2	1.58±0.0	2.86±0.5	2.76±0.0	1.20±0.1	8.76±0.0	11.50±0.2	16.60±6.8	2.89±0.0	4.60±0.3	4.89±0.5	5.51±0.3	12.14±0.2
**10**	Ferulic acid	6.13±1.5	6.38±3.7	4.94±0.5	13.56±0.4	6.81±1.2	5.75±0.5	0.66±0.0	2.13±0.0	0.30±0.2	0.85±0.0	5.66±0.0	13.89±0.5	20.81±9.8	4.75±0.0	2.72±0.2	3.12±0.7	2.98±0.1	8.37±0.2
**11**	3,5-Dicaffeoylquinic acid	0.00±0.0	0.00±0.0	1.09±0.1	0.01±0.0	0.05±0.0	0.00±0.0	0.01±0.0	0.00±0.0	0.09±0.0	0.00±0.0	3.03±0.0	0.01±0.0	0.050.0	0.01±0.0	3.98±0.2	4.38±0.4	3.97±0.3	0.61±0.0
**12**	Ellagic acid	0.09±0.0	1.67±0.3	5.08±0.3	3.58±0.2	2.20±0.1	0.84±0.1	3.35±0.1	1.38±0.6	0.59±0.0	0.82±0.1	9.24±0.0	7.43±0.2	8.80±2.6	0.74±0.0	7.50±0.2	10.19±2.5	17.49±1.3	11.19±0.6
	**Flavonoids:**																		
	**Anthocyanins TOT**	**0.00**	**0.00**	**0.00**	**0.00**	**0.06**	**0.20**	**0.00**	**0.00**	**0.00**	**0.00**	**0.00**	**0.00**	**0.00**	**0.00**	**0.00**	**0.00**	**0.00**	**0.00**
**13**	Delphinidin 3,5 diglucoside	0.00±0.0	0.00±0.0	0.00±0.0	0.00±0.0	0.00±0.0	0.00±0.0	0.00±0.0	0.00±0.0	0.00±0.0	0.00±0.0	0.00±0.0	0.00±0.0	0.00±0.0	0.00±0.0	0.00±0.0	0.00±0.0	0.00±0.0	0.00±0.0

14	Delphinidin-3-galactoside	0.00±0.0	0.00±0.0	0.00±0.0	0.00±0.0	0.00±0.0	0.00±0.0	0.00±0.0	0.00±0.0	0.00±0.0	0.00±0.0	0.00±0.0	0.00±0.0	0.00±0.0	0.00±0.0	0.00±0.0	0.00±0.0	0.00±0.0	0.00±0.0
15	Cyanidin-3-glucoside	0.00±0.0	0.00±0.0	0.00±0.0	0.00±0.0	0.00±0.0	0.00±0.0	0.00±0.0	0.00±0.0	0.00±0.0	0.00±0.0	0.00±0.0	0.00±0.0	0.00±0.0	0.00±0.0	0.00±0.0	0.00±0.0	0.00±0.0	0.00±0.0
16	Petunidin-3-glucoside	0.00±0.0	0.00±0.0	0.00±0.0	0.00±0.0	0.06±0.0	0.16±0.06	0.00±0.0	0.00±0.0	0.00±0.0	0.00±0.0	0.00±0.0	0.00±0.0	0.00±0.0	0.00±0.0	0.00±0.0	0.00±0.0	0.00±0.0	0.00±0.0
17	Pelargonidin-3-rutinoside	0.00±0.0	0.00±0.0	0.00±0.0	0.00±0.0	0.00±0.0	0.00±0.0	0.00±0.0	0.00±0.0	0.00±0.0	0.00±0.0	0.00±0.0	0.00±0.0	0.00±0.0	0.00±0.0	0.00±0.0	0.00±0.0	0.00±0.0	0.00±0.0
18	Pelargonidin-3-glucoside	0.00±0.0	0.00±0.0	0.00±0.0	0.00±0.0	0.00±0.0	0.00±0.0	0.00±0.0	0.00±0.0	0.00±0.0	0.00±0.0	0.00±0.0	0.00±0.0	0.00±0.0	0.00±0.0	0.00±0.0	0.00±0.0	0.00±0.0	0.00±0.0
19	Malvidin-3-galactoside	0.00±0.0	0.00±0.0	0.00±0.0	0.00±0.0	0.00±0.0	0.00±0.0	0.00±0.0	0.00±0.0	0.00±0.0	0.00±0.0	0.00±0.0	0.00±0.0	0.00±0.0	0.00±0.0	0.00±0.0	0.00±0.0	0.00±0.0	0.00±0.0
	**Flavonols TOT**	**0.22**	**3.18**	**8.42**	**5.59**	**4.92**	**2.53**	**4.95**	**2.84**	**0.91**	**3.15**	**12.05**	**12.11**	**13.65**	**1.25**	**11.05**	**12.43**	**13.73**	**9.00**
20	Rutin	0.00±0.0	0.03±0.0	0.07±0.0	0.13±0.0	0.10±0.0	0.21±0.0	0.16±0.0	0.12±0.0	0.04±0.0	0.04±0.0	0.39±0.0	0.27±0.0	0.28±0.1	0.06±0.0	0.25±0.0	0.32±0.0	0.32±0.0	0.28±0.0
21	Isoquercitrin	0.00±0.0	0.02±0.0	0.11±0.0	0.05±0.0	0.05±0.0	0.02±0.0	0.16±0.0	0.06±0.0	0.10±0.0	0.14±0.0	0.24±0.0	0.60±0.0	1.06±0.5	0.02±0.0	0.11±0.0	0.11±0.0	0.09±0.0	0.22±0.0
22	Quercitrin	0.01±0.0	0.03±0.0	0.25±0.0	0.05±0.0	0.09±0.0	0.03±0.0	0.38±0.0	0.06±0.0	0.02±0.0	0.17±0.0	0.24±0.0	0.64±0.0	1.56±0.9	0.01±0.0	0.07±0.0	0.15±0.0	0.09±0.0	0.40±0.0
23	Myricetin	0.00±0.0	0.02±0.0	0.16±0.0	0.03±0.0	0.03±0.0	0.02±0.0	0.20±0.0	0.03±0.0	0.00±0.0	0.08±0.0	0.12±0.0	0.04±0.0	0.02±0.0	0.01±0.0	0.32±0.0	0.48±0.0	0.97±0.1	0.12±0.0
24	Kaempferol-3-glucoside	0.00±0.0	0.19±0.0	0.24±0.0	0.11±0.0	0.03±0.0	0.01±0.0	0.08±0.0	0.01±0.0	0.01±0.0	0.02±0.0	0.10±0.0	0.41±0.1	0.35±0.1	0.06±0.0	0.53±0.0	0.48±0.0	0.48±0.0	0.11±0.0
25	Quercetin	0.08±0.0	1.42±0.2	4.94±0.3	2.50±0.2	2.32±0.0	0.83±0.0	2.76±0.3	1.31±0.2	0.59±0.0	1.38±0.0	8.55±0.0	5.88±0.1	7.07±2.7	0.74±0.0	7.91±0.2	8.70±0.7	9.62±0.5	4.62±0.0
26	Isorhamnetin	0.13±0.0	1.40±0.0	2.26±0.1	2.68±0.1	2.19±0.1	1.38±0.1	1.16±0.0	1.22±0.2	0.14±0.0	1.28±0.0	2.22±0.0	3.85±0.4	3.13±0.5	0.33±0.0	1.46±0.0	1.71±0.1	1.67±0.1	2.97±0.0
27	Hyperoside	0.00±0.0	0.00±0.0	0.02±0.0	0.01±0.0	0.02±0.0	0.01±0.0	0.05±0.0	0.01±0.0	0.03±0.0	0.04±0.0	0.07±0.0	0.14±0.0	0.17±0.0	0.00±0.0	0.02±0.0	0.02±0.0	0.02±0.0	0.04±0.0
28	Kaempferol	0.00±0.0	0.06±0.0	0.38±0.0	0.02±0.0	0.10±0.0	0.02±0.0	0.00±0.0	0.02±0.0	0.00±0.0	0±0.0	0.12±0.0	0.28±0.0	0.00±0.0	0.01±0.0	0.37±0.0	0.46±0.0	0.46±0.0	0.24±0.0
	**Flavan-3-ols TOT**	**0.87**	**0.00**	**0.05**	**0.01**	**0.15**	**0.00**	**0.03**	**0.25**	**0.00**	**0.45**	**0.01**	**0.00**	**0.13**	**0.03**	**0.00**	**0.01**	**0.00**	**0.27**
29	Catechin	0.00±0.0	0.00±0.0	0.02±0.0	0.00±0.0	0.02±0.0	0.00±0.0	0.00±0.0	0.00±0.0	0.00±0.0	0.00±0.0	0.00±0.0	0.00±0.0	0.13±0.2	0.00±0.0	0.00±0.0	0.00±0.0	0.00±0.0	0.07±0.0
30	Epicatechin	0.87±0.4	0±0.0	0.01±0.0	0.00±0.0	0.11±0.0	0±0.0	0.02±0.0	0.24±0.1	0±0.0	0.45±0.0	0.01±0.0	0±0.0	0±0.0	0±0.0	0.00±0.0	0.01±0.0	0±0.0	0.18±0.0

31	Procyanidin B2	0.00±0.0	0.00±0.0	0.00±0.0	0.00±0.0	0.00±0.0	0.00±0.0	0.01±0.0	0.00±0.0	0.00±0.0	0.00±0.0	0.00±0.0	0.00±0.0	0.00±0.0	0.00±0.0	0.00±0.0	0.00±0.0	0.00±0.0	0.00±0.0
32	Procyanidin A2	0.00±0.0	0.00±0.0	0.03±0.0	0.01±0.0	0.02±0.0	0.00±0.0	0.00±0.0	0.02±0.0	0.00±0.0	0.00±0.0	0.00±0.0	0.00±0.0	0.00±0.0	0.03±0.0	0.00±0.0	0.00±0.0	0.00±0.0	0.03±0.0
	**Dihydro-chalcones TOT**	**0.00**	**0.00**	**0.00**	**0.00**	**0.00**	**0.00**	**0.00**	**0.00**	**0.00**	**0.00**	**0.00**	**0.00**	**0.00**	**0.00**	**0.00**	**0.00**	**0.00**	**0.00**
33	Phloridzin	0.00±0.0	0.00±0.0	0.00±0.0	0.00±0.0	0.00±0.0	0.00±0.0	0.00±0.0	0.00±0.0	0.00±0.0	0.00±0.0	0.00±0.0	0.00±0.0	0.00±0.0	0.00±0.0	0.00±0.0	0.00±0.0	0.00±0.0	0.00±0.0
34	Phloretin	0.00±0.0	0.00±0.0	0.00±0.0	0.00±0.0	0.00±0.0	0.00±0.0	0.00±0.0	0.00±0.0	0.00±0.0	0.00±0.0	0.00±0.0	0.00±0.0	0.00±0.0	0.00±0.0	0.00±0.0	0.00±0.0	0.00±0.0	0.00±0.0
	**Flavanones TOT**	**0.00**	**0.00**	**0.15**	**0.01**	**0.00**	**0.00**	**1.14**	**0.12**	**0.00**	**0.02**	**0.00**	**0.00**	**0.00**	**0.00**	**0.00**	**0.00**	**0.00**	**0.08**
35	Hesperidin	0.00±0.0	0.00±0.0	0.02±0.0	0.00±0.0	0.00±0.0	0.00±0.0	0.01±0.0	0.01±0.0	0.00±0.0	0.00±0.0	0.00±0.0	0.00±0.0	0.00±0.0	0.00±0.0	0.00±0.0	0.00±0.0	0.00±0.0	0.00±0.0
36	Naringin	0.00±0.0	0.00±0.0	0.13±0.0	0.01±0.0	0.00±0.0	0.00±0.0	1.13±0.2	0.11±0.0	0.00±0.0	0.02±0.0	0.00±0.0	0.00±0.0	0.00±0.0	0.00±0.0	0.00±0.0	0.00±0.0	0.00±0.0	0.08±0.0
	**Non-phenolic acids TOT**	**0.59**	**0.04**	**0.05**	**0.16**	**0.24**	**0.16**	**0.04**	**0.49**	**0.00**	**0.03**	**0.05**	**0.15**	**0.10**	**0.03**	**0.03**	**0.04**	**0.03**	**0.15**
37	*Trans*-cinnamic acid	0.59±0.0	0.04±0.0	0.05±0.0	0.16±0.0	0.24±0.0	0.16±0.0	0.04±0.0	0.49±0.0	0.00±0.0	0.03±0.0	0.05±0.0	0.15±0.0	0.10±0.0	0.03±0.0	0.03±0.0	0.04±0.0	0.03±0.0	0.15±0.0
	**Total phenolic content**	**59.36**	**54.65**	**100.65**	**94.52**	**76.25**	**41.29**	**44.85**	**52.34**	**19.56**	**28.25**	**82.60**	**161.68**	**243.94**	**34.45**	**96.55**	**115.08**	**138.95**	**123.44**

Table S4: Volatile compounds (%) in honey samples.

**Compound**		**RI**	**H1**	**H2**	**H3**	**H4**	**H5**	**H6**	**H7**	**H8**	**H9**	**H10**	**H11**	**H12**	**H13**	**H14**	**H15**	**H16**	**H17**	**H18**
Ketones																				
**1**	Acetoin	708	n.d.	n.d.	n.d.	n.d.	n.d.	n.d.	n.d.	n.d.	n.d.	n.d.	n.d.	n.d.	n.d.	n.d.	n.d.	n.d.	6.72±0.41	n.d.
**2**	Acetophenone	1055	5.54±0.09	n.d.	n.d.	n.d.	n.d.	n.d.	n.d.	4.80±0.15	n.d.	n.d.	n.d.	n.d.	n.d.	n.d.	n.d.	n.d.	n.d.	n.d.

**3**	2,6,6-Trimethyl-2-cyclohexene-1,4-dione	1132	n.d.	n.d.	n.d.	n.d.	n.d.	n.d.	1.04 ±0.16	n.d.	n.d.	12.02 ±0.19	n.d.	n.d.	n.d.	n.d.	n.d.	4.57 ±0.87	n.d.	n.d.
Alcohols																				
**4**	3-Buten-1-ol,3-methyl	727	n.d.	n.d.	4.59 ±0.04	n.d.	n.d.	n.d.	0.22 ±0.04	3.17 ±0.03	n.d.	n.d.	3.97 ±0.10	2.43 ±0.41	2.31 ±0.37	8.34 ±	n.d.	n.d.	n.d.	1.10 ±0.02
**5**	1-Butanol,2-methyl	734	n.d.	n.d.	n.d.	n.d.	n.d.	n.d.	n.d.	n.d.	n.d.	n.d.	n.d.	n.d.	n.d.	n.d.	n.d.	n.d.	n.d.	1.06 ±0.01
**6**	2,3-butanediol	787	n.d.	n.d.	4.84 ±0.05	n.d.	n.d.	n.d.	0.19 ±1.24	n.d.	n.d.	8.78 ±0.16	n.d.	n.d.	n.d.	n.d.	n.d.	n.d.	n.d.	1.56 ±0.01
**7**	Benzyl alcohol	1025	16.90 ±0.63	n.d.	n.d.	n.d.	3.92 ±1.05	11.31 ±0.92	n.d.	5.82 ±0.45	n.d.	n.d.	n.d.	n.d.	2.02 ±0.31	n.d.	n.d.	n.d.	n.d.	n.d.
**8**	Linalool oxide	1062	n.d.	8.12 ±0.61	7.07 ±0.50	n.d.	2.68 ±0.07	n.d.	n.d.	4.66 ±0.06	n.d.	n.d.	16.70 ±1.80	n.d.	n.d.	n.d.	8.62 ±0.75	11.54 ±0.79	10.89 ±0.03	n.d.
**9**	1-Octanol	1063	n.d.	n.d.	n.d.	n.d.	n.d.	n.d.	n.d.	n.d.	n.d.	n.d.	n.d.	n.d.	n.d.	n.d.	n.d.	n.d.	n.d.	9.86 ±1.76
**10**	Linalool	1092	n.d.	3.55 ±0.49	5.82 ±0.42	n.d.	n.d.	n.d.	n.d.	n.d.	n.d.	n.d.	n.d.	5.42 ±0.17	7.15 ±0.21	n.d.	n.d.	n.d.	n.d.	4.28 ±0.61
**11**	1,5,7-Octatrien-3-ol,3,7-dimethyl	1097	n.d.	n.d.	n.d.	40.00	n.d.	n.d.	n.d.	n.d.	n.d.	n.d.	n.d.	37.90 ±1.15	n.d.	n.d.	20.40 ±2.41	n.d.	n.d.	n.d.

**12**	Phenylethyl alcohol	1103	7.85 ±0.39	13.87 ±0.34	8.14 ±0.11	8.42 ±0.02	8.49 ±0.75	7.35 ±0.06	2.34 ±0.44	6.59 ±0.01	n.d.	8.21 ±0.00	5.57 ±0.35	5.49 ±0.29	4.61 ±0.18	55.87 ±3.41	11.86 ±1.95	9.01 ±0.75	10.68 ±0.10	1.64 ±0.30
**13**	p-Mentha-trans-2,8-dien-1-ol	1123	n.d.	n.d.	n.d.	n.d.	n.d.	6.32 ±0.05	n.d.	n.d.	n.d.	n.d.	n.d.	n.d.	n.d.	n.d.	n.d.	n.d.	n.d.	n.d.
**14**	Terpinen-4-ol	1163	n.d.	n.d.	n.d.	n.d.	n.d.	7.11 ±0.18	n.d.	n.d.	n.d.	n.d.	n.d.	n.d.	n.d.	n.d.	n.d.	n.d.	n.d.	n.d.
**15**	Benzenemethanol,α,α,4-trimethyl	1173	n.d.	n.d.	n.d.	n.d.	n.d.	n.d.	n.d.	n.d.	n.d.	n.d.	n.d.	n.d.	n.d.	n.d.	n.d.	n.d.	n.d.	1.96 ±0.17
**16**	p-Cymen-8-ol	1173	n.d.	n.d.	n.d.	n.d.	n.d.	12.79 ±0.84	n.d.	n.d.	n.d.	n.d.	n.d.	n.d.	n.d.	n.d.	n.d.	n.d.	n.d.	n.d.
**17**	α-Terpineol	1177	n.d.	n.d.	n.d.	n.d.	n.d.	n.d.	n.d.	n.d.	n.d.	n.d.	n.d.	n.d.	n.d.	n.d.	5.77 ±0.41	n.d.	n.d.	n.d.
**18**	1-Dodecanol	1471	n.d.	n.d.	n.d.	n.d.	n.d.	n.d.	n.d.	n.d.	0.87 ±	n.d.	n.d.	n.d.	n.d.	n.d.	n.d.	n.d.	n.d.	n.d.
Aldehydes																				
**19**	2-Butenal,2-methyl	739	n.d.	n.d.	n.d.	n.d.	n.d.	n.d.	0.06 ±0.05	n.d.	n.d.	n.d.	n.d.	1.29 ±	n.d.	n.d.	n.d.	n.d.	n.d.	1.07 ±0.01
**20**	Hexanal	800	n.d.	n.d.	n.d.	n.d.	n.d.	n.d.	0.09 ±0.7	n.d.	n.d.	n.d.	n.d.	n.d.	n.d.	n.d.	n.d.	n.d.	n.d.	n.d.
**21**	Heptanal	900	n.d.	n.d.	n.d.	n.d.	n.d.	n.d.	0.50 ±1.41	n.d.	n.d.	n.d.	n.d.	n.d.	1.52 ±0.28	n.d.	n.d.	n.d.	n.d.	1.05
**22**	Benzaldeh	950	16.49 ±2.3	43.44 ±1.9	13.78 ±0.0	28.99 ±0.7	16.00 ±2.7	9.98 ±0.5	n.d.	15.10 ±1.5	0.78 ±0.3	8.19 ±0.0	8.65 ±0.1	13.17 ±0.2	5.53 ±0.0	20.46 ±2.0	6.10 ±0.4	14.69 ±1.4	17.78 ±1.2	7.89 ±0.0

	yde		0	2	7	3	4	2		3	2	3	7	9	9	5	5	7	3	6
**23**	Octanal	999	n.d.	n.d.	n.d.	n.d.	n.d.	n.d.	1.89 ±0.31	n.d.	n.d.	n.d.	n.d.	n.d.	5.78 ±	n.d.	n.d.	n.d.	n.d.	6.54 ±1.07
**24**	Benzeneacetaldehyde	1033	n.d.	2.04 ±0.7	6.26 ±0.03	4.13 ±0.18	4.22 ±0.27	n.d.	63.53 ±8.01	4.96 ±0.28	n.d.	8.07 ±0.00	11.81 ±2.14	n.d.	n.d.	n.d.	16.13 ±1.29	14.76 ±1.46	23.73 ±0.39	1.28 ±0.01
**25**	Nonanal	1097	n.d.	8.43 ±0.43	n.d.	n.d.	n.d.	16.22 ±1.25	17.12 ±2.81	n.d.	n.d.	8.12 ±0.02	n.d.	n.d.	36.06 ±5.01	n.d.	n.d.	10.97 ±0.75	n.d.	30.28 ±4.53
**26**	Lilac aldehyde A	1145	n.d.	n.d.	7.69 ±0.62	n.d.	5.33 ±0.11	n.d.	n.d.	n.d.	n.d.	n.d.	3.67 ±0.05	8.88 ±	8.50 ±	n.d.	n.d.	n.d.	n.d.	6.48 ±0.50
**27**	Decanal	1197	n.d.	n.d.	n.d.	n.d.	n.d.	n.d.	n.d.	n.d.	n.d.	8.19 ±0.03	6.70 ±0.48	n.d.	n.d.	n.d.	n.d.	n.d.	n.d.	1.85 ±0.11
**28**	Cinnamaldehyde	1254	5.72 ±0.49	n.d.	n.d.	n.d.	n.d.	n.d.	n.d.	9.24 ±0.57	n.d.	n.d.	n.d.	n.d.	n.d.	n.d.	n.d.	n.d.	n.d.	n.d.
**29**	Benzaldehyde,3,4,5-trimethoxy	1597	n.d.	n.d.	n.d.	n.d.	n.d.	n.d.	n.d.	n.d.	n.d.	n.d.	n.d.	1.65 ±0.01	2.17 ±0.78	n.d.	n.d.	n.d.	n.d.	n.d.
Furanones																				
**30**	3(2H)-furanone,dihydro-2-methyl	805	n.d.	n.d.	n.d.	n.d.	n.d.	n.d.	n.d.	3.14 ±0.02	n.d.	n.d.	n.d.	n.d.	n.d.	n.d.	n.d.	n.d.	n.d.	n.d.
Esters																				
**31**	Butanoic acid-3-mehtyl	832	6.04 ±2.35	n.d.	n.d.	n.d.	n.d.	n.d.	n.d.	n.d.	n.d.	n.d.	n.d.	n.d.	n.d.	n.d.	n.d.	n.d.	n.d.	n.d.
**32**	Butanoic	844	7.00 ±0.7	n.d.	n.d.	n.d.	2.65 ±0.1	n.d.	n.d.	n.d.	n.d.	n.d.	n.d.	n.d.	n.d.	n.d.	n.d.	n.d.	n.d.	n.d.

	acid, 2-methyl		5																	
**33**	Pentanoic acid, 3-methyl	938	n.d.	2.66 ±0.29	n.d.	n.d.	11.41 ±1.59	n.d.	n.d.	n.d.	n.d.	n.d.	n.d.	n.d.	n.d.	n.d.	n.d.	n.d.	n.d.	n.d.
**34**	Hexanoic acid,2-ethyl	1119	n.d.	n.d.	6.82 ±0.32	n.d.	n.d.	n.d.	n.d.	n.d.	n.d.	n.d.	n.d.	n.d.	n.d.	n.d.	n.d.	n.d.	n.d.	n.d.
**35**	Butanoic acid,3-hexenyl ester	1180	4.07 ±0.05	n.d.	5.89 ±0.03	1.70 ±0.26	3.21 ±0.16	n.d.	0.80 ±0.09	n.d.	n.d.	n.d.	n.d.	n.d.	n.d.	n.d.	n.d.	n.d.	n.d.	n.d.
Pyrazines																				
**36**	Pyrazine,2,6-dimethyl	906	n.d.	n.d.	n.d.	n.d.	n.d.	n.d.	n.d.	n.d.	n.d.	n.d.	n.d.	n.d.	n.d.	n.d.	n.d.	n.d.	n.d.	5.35 ±0.86
**37**	Pyrazine, trimethyl	996	n.d.	n.d.	n.d.	n.d.	n.d.	n.d.	n.d.	n.d.	n.d.	n.d.	n.d.	1.88 ±	2.93 ±	n.d.	n.d.	n.d.	n.d.	2.99 ±0.37
Furans																				
**38**	Furfural	827	4.00 ±0.04	5.66 ±0.05	15.84 ±0.62	2.31 ±0.39	4.69 ±0.44	7.46 ±0.07	0.77 ±0.13	15.96 ±2.23	62.49 ±40.83	8.30 ±0.05	n.d.	1.95 ±0.01	1.35 ±0.51	5.67 ±0.47	3.91 ±0.12	4.80 ±0.30	14.21 ±1.91	2.13 ±0.15
**39**	2-Acetylfuran	908	n.d.	n.d.	4.80 ±0.15	n.d.	n.d.	n.d.	0.16 ±0.52	5.50 ±0.28	n.d.	8.08 ±0.01	1.92 ±0.10	n.d.	n.d.	n.d.	3.38 ±0.38	3.70 ±0.10	4.96 ±0.07	n.d.
**40**	2-furancarboxaldehyde, 5-	955	n.d.	n.d.	n.d.	n.d.	n.d.	n.d.	n.d.	n.d.	0.68 ±0.44	n.d.	n.d.	n.d.	n.d.	n.d.	n.d.	n.d.	n.d.	n.d.

	methyl																			
	Acids																			
**41**	Hexanoic acid	977	3.72 ±0.44	n.d.	n.d.	n.d.	n.d.	n.d.	n.d.	4.47 ±0.28	n.d.	n.d.	n.d.	n.d.	n.d.	n.d.	n.d.	n.d.	n.d.	n.d.
**42**	Octanoic acid	1172	6.29 ±0.55	n.d.	n.d.	n.d.	2.86 ±	n.d.	n.d.	3.88 ±1.25	12.65 ±0.05	n.d.	7.90 ±2.30	2.36 ±0.07	1.92 ±0.20	n.d.	9.08 ±0.41	6.93 ±0.73	n.d.	2.19 ±0.03
**43**	Benzeneacetic acid	1241	n.d.	n.d.	n.d.	n.d.	n.d.	n.d.	n.d.	n.d.	n.d.	n.d.	n.d.	n.d.	n.d.	n.d.	4.66 ±	n.d.	n.d.	n.d.
**44**	Nonanoic acid	1269	10.81 ±0.32	4.79 ±0.73	6.09 ±1.00	2.88 ±0.98	21.32 ±3.08	n.d.	1.09 ±0.21	4.44 ±0.26	6.61 ±1.30	n.d.	12.80 ±2.10	2.80 ±0.02	3.62 ±0.92	n.d.	n.d.	4.25 ±0.26	n.d.	n.d.
Terpenes																				
**45**	Thymol	1283	n.d.	n.d.	n.d.	n.d.	3.17 ±0.12	11.53 ±0.09	n.d.	n.d.	1.00 ±	8.07 ±0.00	5.88 ±0.90	n.d.	n.d.	n.d.	n.d.	n.d.	n.d.	n.d.
**% Identified**			**94.43**	**92.56**	**97.63**	**88.43**	**89.95**	**90.07**	**89.8**	**91.73**	**85.08**	**86.03**	**85.57**	**85.22**	**85.47**	**90.34**	**89.91**	**85.22**	**88.97**	**90.56**
